# Experience of targeted Usher exome sequencing as a clinical test

**DOI:** 10.1002/mgg3.25

**Published:** 2013-07-10

**Authors:** Thomas Besnard, Gema García-García, David Baux, Christel Vaché, Valérie Faugère, Lise Larrieu, Susana Léonard, Jose M Millan, Sue Malcolm, Mireille Claustres, Anne-Françoise Roux

**Affiliations:** 1 U827, InsermMontpellier, F-34000, France; 2 Univ, Montpellier IMontpellier, F-34000, France; 3 Grupo de Investigación en Enfermedades Neurosensoriales, Instituto de Investigación Sanitaria IIS-La Fe and CIBERERValencia, Spain; 4 Laboratoire de Génétique Moléculaire, CHU MontpellierMontpellier, F-34000, France; 5 Clinical and Molecular Genetics, Institute of Child Health, University College LondonLondon, United Kingdom

**Keywords:** Bioinformatics, next-generation sequencing, NSHL, Usher syndrome, variant prioritization

## Abstract

We show that massively parallel targeted sequencing of 19 genes provides a new and reliable strategy for molecular diagnosis of Usher syndrome (USH) and nonsyndromic deafness, particularly appropriate for these disorders characterized by a high clinical and genetic heterogeneity and a complex structure of several of the genes involved. A series of 71 patients including Usher patients previously screened by Sanger sequencing plus newly referred patients was studied. Ninety-eight percent of the variants previously identified by Sanger sequencing were found by next-generation sequencing (NGS). NGS proved to be efficient as it offers analysis of all relevant genes which is laborious to reach with Sanger sequencing. Among the 13 newly referred Usher patients, both mutations in the same gene were identified in 77% of cases (10 patients) and one candidate pathogenic variant in two additional patients. This work can be considered as pilot for implementing NGS for genetically heterogeneous diseases in clinical service.

## Introduction

Usher syndrome (USH) is an autosomal recessive disorder with a prevalence of at least 5/100,000 characterized by the association of sensorineural hearing loss (HL) and visual impairment due to retinitis pigmentosa (RP). USH is the most common form of deaf–blindness (Saihan et al. 2009). Three clinical subtypes (USH1, USH2, and USH3) are distinguished depending on the severity and progression of HL and presence or absence of vestibular areflexia and this distinction is generally used to guide molecular diagnosis. USH1 is the most severe form with congenital profound HL and vestibular areflexia. USH2 is the most common clinical form of the disorder, accounting for over a half of USH cases and is characterized by congenital moderate-to-severe HL, with normal vestibular function. In USH3, the HL is progressive with variable vestibular function. USH3 is rare except in some populations with founder effects where it is responsible for more than 40% of the Finnish and Jewish Ashkenazi USH cases (Saihan et al. 2009).

Clinical heterogeneity is accompanied by high genetic heterogeneity. To date 11 genes responsible for the disease are known. Five USH1 genes have been extensively studied: *MYO7A* (USH1B), *CDH23* (USH1D), *PCDH15* (USH1F), *USH1C* (USH1C), and *USH1G* (USH1G), with mutations in *MYO7A* being the most prevalent (Roux et al. 2011; Le Quesne Stabej et al. 2012). A sixth gene, *CIB2* (USH1J), has been very recently reported in a single family (Riazuddin et al. 2012). Among the three identified USH2 genes, *USH2A* (USH2A), *GPR98* (USH2C), and *DFNB31* (USH2D), *USH2A* mutations have been shown to be responsible for 70–80% of USH2 cases (Besnard et al. 2012; Le Quesne Stabej et al. 2012). Until recently, *CLRN1* (USH3A) was the only gene known responsible for USH3 (Joensuu et al. 2001), but the *HARS* gene was recently proposed as a novel USH3 gene (Puffenberger et al. 2012). In addition, a twelfth gene, *PDZD7*, contributes to USH2 as a modifier of the retinal phenotype on a *USH2A* background or in digenic inheritance with *GPR98* (Ebermann et al. 2010). Multiple isoforms have been described for most of these genes of which, *USH1C*, *PCDH15*, *USH2A* isoforms have been well characterized (Bitner-Glindzicz et al. 2000; Verpy et al. 2000; van Wijk et al. 2004; Ahmed et al. 2008).

Mutations in *MYO7A*, *USH1C*, *CDH23, PCDH15, DFNB31,* and *CIB2* can also cause nonsyndromic hearing loss (NSHL) and mutations in *USH2A* and *CLRN1* give rise to isolated autosomal recessive RP (see retinal and hearing impairment genetic mutation database, which includes USHbases and other NSHL genes: https://grenada.lumc.nl/LOVD2/Usher_montpellier/). Recently, a mutation in the short isoform of *USH1C* has been shown to be associated with RP and late-onset deafness (Khateb et al. 2012).

Molecular genetic diagnosis for USH has developed from the scanning of restricted portions of USH genes (Adato et al. 1997) to extensive direct sequencing (Aller et al. 2006; Roux et al. 2006, 2011; Baux et al. 2007; Dreyer et al. 2008; Bonnet et al. 2011; Garcia-Garcia et al. 2011; Besnard et al. 2012; Le Quesne Stabej et al. 2012). Because of the genetic heterogeneity, prioritization of the genes to be sequenced was achieved by preliminary linkage analysis (Roux et al. 2006, 2011). Due to the large size of most Usher genes (in total more than 350 exons), Sanger sequencing of genes one-by-one remains expensive and time consuming. Furthermore, large rearrangements have been described in *MYO7A, CDH23, GPR98, USH2A* and, particularly, in *PCDH15,* and their detection requires array-CGH studies and/or multiplex ligation-dependent probe amplification (see USHbases). Taken together, these strategies allow a reliable diagnosis for Usher patients with a mutation detection rate of about 90% for USH1 and USH2 patients (Roux et al. 2011; Besnard et al. 2012). A genotyping microarray commercially available (Cremers et al. 2006) allows rapid screening for hundreds of previously identified variations in nine USH genes (Vozzi et al. 2011), but its application in clinical diagnosis is hampered by a very low detection rate as most USH-causing DNA alterations are private or restricted to one or two families (see USHBases).

NGS technology has recently demonstrated its capacity to detect DNA variants in sensorineural disorders known to be genetically heterogeneous (Brownstein et al. 2011; Neveling et al. 2012; Redin et al. 2012), and a targeted NGS protocol on nine samples showed a technical performance compatible with potential use as a diagnostic platform when applied to HL (Shearer et al. 2010). A recent study applied to USH compared two different enrichment methods and reported a higher efficiency in mutation detection using a Long-Range PCR targeted approach compared to whole-exome capture (Licastro et al. 2012).

We have designed an NGS-based workflow using a solution-based capture method, which we applied to 71 patients with the aim of rigorously evaluating the feasibility of NGS for screening Usher genes in a clinical diagnostic setting. Forty-seven Usher patients (test sample), either negative for USH gene mutations or carrying a single mutation after Sanger sequencing and array-CGH analyses, were used as a test cohort to establish criteria and thresholds for accurate generation and filtering of the data, as well as prioritization and annotation of the variants, and calculation of analytical sensitivity. The validated protocol was then applied to 13 newly referred Usher patients (Usher Diagnosis Group). We also included 11 NSHL patients as mutations in the targeted genes have been found albeit only accounting for a minority of cases.

## Material and Methods

### Patients

A total of 71 subjects (21 Spanish and 50 French), classified by their clinical history and ophthalmologic, audiometric and vestibular tests, were enrolled in this study (Fig. S1). Audiograms from patients presenting with NSHL were collected and profound HL confirmed. The local Ethics Committee approved molecular analyses and consent to genetic testing was obtained from adult probands or parents in the case of minors. DNA was extracted from blood samples and quality and quantity assessed using standard techniques.

### Test sample

Among the 47 patients included in this group, seven were classified as USH1, 34 as USH2, and two as USH3 (Table S1). Four of them could not be classified because of lack of clinical data. All patients had previously been studied for at least one Usher gene by Sanger sequencing (Table S1) which had led to the identification of one putative causative mutation in 22 of them. The remaining 25 patients had no identified pathogenic mutation in any of the genes screened by Sanger sequencing.

### Diagnostic sample

#### Usher diagnosis group

Thirteen patients were included in this group. No molecular study had been performed prior to NGS. Five of them were considered to be USH1, seven to be USH2, and one to be USH3.

#### NSHL diagnosis group

Eleven patients presenting with NSHL were selected. A genetic origin of deafness was suspected based on the absence of any environmental or infectious cause, presence of familial cases or documented consanguinity. All had been previously screened for mutations at the DFNB1 locus and one of them was a *GJB2* c.35delG heterozygote.

### Sequence capture and sequencing

A custom solution-based sequence capture manufactured by Roche Nimblegen (Madison, WI) (SeqCap EZ Choice Library) included a total of 634 exons (and 100 bp of the flanking intronic regions) from 19 genes (nine known Usher genes, two candidate Usher genes [*PDZD7* and *VEZT*], seven NSHL genes [*GJB2, GJB6, GJB3, MYO15A, TECTA, OTOF, TMC1*], and *CHM* [*REP-1*] gene), and their 5′ and 3′ untranslated regions. All annotated transcripts were included (Table S2). The design included the intronic *USH2A* region encompassing the pseudoexon recently described (Vaché et al. 2012). The entire custom design spanned 326 kb. The final capture size was 364 kb covered by more than 32,000 different biotinylated probes. By merging the overlapping regions, the design encompassed 535 different regions.

Sequence capture was performed according to the User's Guide “NimbleGen SeqCap EZ Library LR” (Version 2.0, November 2011). DNA libraries were prepared following the instructions from the manufacturer (GS FLX Titanium Rapid Library Preparation Method Manual, January 2010). Genomic DNA (500 ng) was sheared by fragmentation (with a majority of fragments between 400 and 700 bp). Fragments were end repaired, A-tailed, and ligated to the adapters. Small fragments were removed using Agencourt AMPure XP beads (Beckman Coulter, Agencourt, Beverly, MA). The libraries were amplified for 12 cycles by precaptured ligation-mediated polymerase chain reaction (precapture LM-PCR) with primers specific for the adaptors. The amplified libraries were then hybridized to the designed biotinylated probes for 66–72 h at 47°C. The biotinylated probes-DNA hybrids were purified with streptavidin-conjugated magnetic beads and washed. Finally, the captured DNA fragments were eluted/recovered and amplified for 15 cycles (postcapture LM-PCR). The final concentration of each captured library was calculated with a Qubit fluorometer and diluted at 10^7^ molecules/μL. Emulsion PCRs were performed according to the manufacturer's instructions (emPCR Amplification Method Manual Lib-L GS Junior Titanium Series, May 2010, Rev. April 2011). The ratio 1 molecule per bead was chosen as input to perform the emPCRs. Sequencing of each library was carried out on Roche GS Junior sequencer according to the manufacturer's protocol (Sequencing Method Manual GS Junior Titanium Series, May 2010, Rev. June 2010).

### Bioinformatics pipeline and prioritization

#### Assembly, coverage, and variant calling

Sequence reads were mapped against the human chromosomes reference (hg19) using the GS Reference Mapper software (Roche, version 2.6 and 2.7). Average depth of coverage (aDOC) for each region was calculated by dividing the sum of the DOC per base within the specific regions by the total region size (in base pairs).


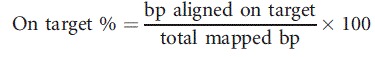


The percentage of *on target* was defined as a ratio between the number of bases aligned in targeted regions and the number of bases mapped in total.


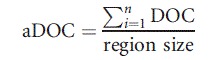


Artifact variants were removed following these criteria: (i) Variants detected in less than 20% of total reads; (ii) *Indels* with a coverage >20 reads but with a disequilibrium between number of forward and reverse sequences (Fwd or Rev <10%); (iii) *Indels* distant from exons (more than ±20 intronic flanking nucleotides).

The remaining variants were annotated adding gene name, known polymorphism from dbSNP131, localization in gene, cDNA and protein nomenclature, using either Annovar (Wang et al. 2010) or Mutalyzer (Wildeman et al. 2008).

We developed in-house software called “GS data online treatment” (GSdot), available at https://neuro-2.iurc.montp.inserm.fr/454/ to automate the calculations and filters described above. The input files were initially generated by GS Reference mapper. More details on how to use the software and the different steps can be obtained from the website.

#### Prioritization of variants and determination of pathogenicity

After automatic filtering performed by GSdot, all the annotated variant files generated (one per patient) were merged into a single one for manual prioritization of the variants. Prioritization consisted of keeping any known pathogenic mutation and, for any variant of unknown clinical significance (VUCS), retaining if it had been found in fewer than five DNAs from the test sample, and was localized in exon or within 20 bp intron–exon boundary.

The pathogenicity of the candidate variants was investigated examining the frequency in patients or controls from our internal database, the locus-specific database LOVD-USHbases, the deafness variation database (http://deafnessvariationdatabase.com/), the exome variant server database (EVS, http://evs.gs.washington.edu/EVS/) or the genome variant database (1000 genomes, http://www.1000genomes.org/, and dbSNP, http://www.ncbi.nlm.nih.gov/snp). Specific in silico studies were conducted following our multistep analysis described in (Roux et al. 2011), using the Usher Syndrome Missense Analysis software (USMA, https://neuro-2.iurc.montp.inserm.fr/USMA/) and Human Splicing Finder (HSF [Desmet et al. 2009], http://www.umd.be/HSF/), which includes two distinct algorithms, namely HSF and MaxEnt (Yeo and Burge 2004). In order to assess the impact of missense candidates in non-Usher genes, ortholog and domain alignments were studied from UCSC (http://genome.ucsc.edu/) and Prosite (http://prosite.expasy.org/), respectively.

All likely pathogenic variants were confirmed by Sanger sequencing, and familial segregation analyses were performed whenever possible. The latter contribute to classification of the VUCS as already described (Roux et al. 2011; Baux et al. 2013), from UV1 to UV4, with UV1 being the least likely to be disease causing.

### Ex vivo splicing assay

DNA from U1157 was used as template in a PCR amplification including exons 62 to 65 of *CDH23* with the High Fidelity Phusion Polymerase (Finnzymes, Espoo, Finland). Amplicons were inserted in the pSPL3 exon-trapping vector between the *Not*I and *Xho*I restriction sites and the constructs were transfected in a human retinal pigment epithelial cells line (ARPE-19) as previously described (Guédard-Méreuze et al. 2009). Forty-eight hours after transfection, RNA was extracted with the Nucleospin RNAII kit (Macherey-Nagel, Hoerdt, France). RT-PCR and splicing alterations analyses were carried out as described before (Le Guédard-Mereuze et al. 2010).

## Results

### Raw data quality

**Figure 1:**
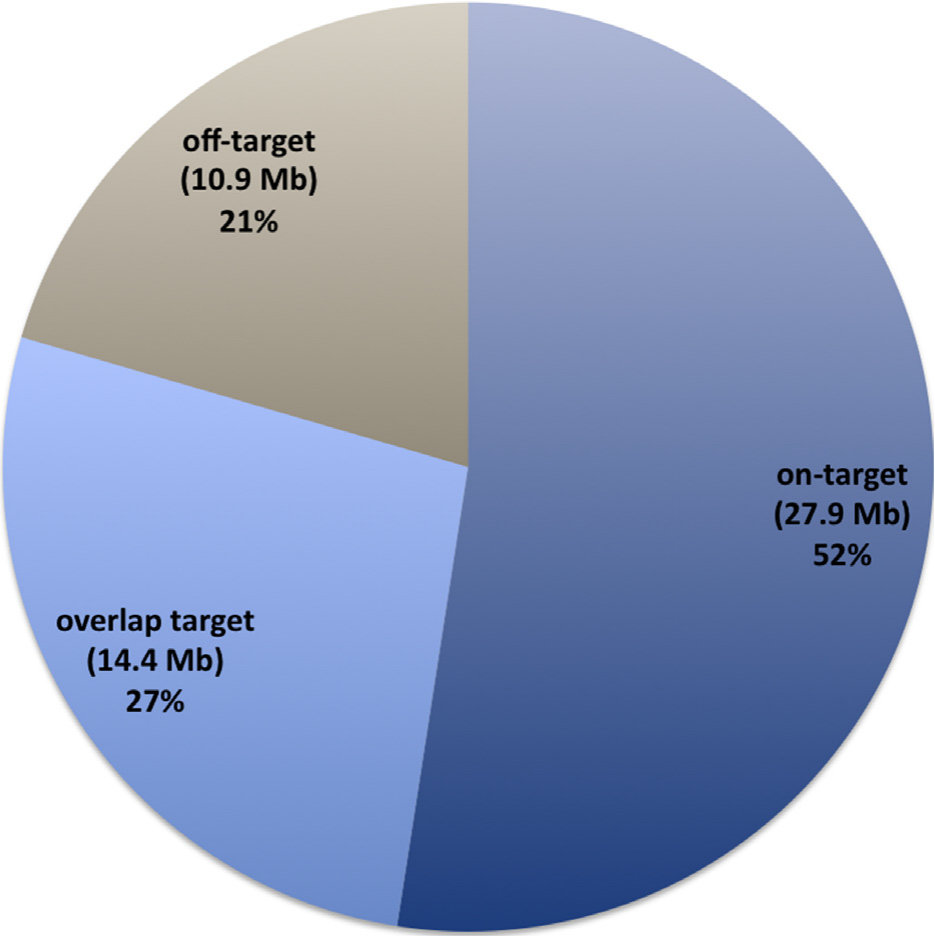
Distribution of mapped bases. The percentages refer to reads mapped to the targeted region (on-target), sequences partially mapped to the targeted region (overlap-target), or reads completely aligned out of region (off-target).

Data obtained from the test sample were used to evaluate the quality of raw data. The number of reads per run was estimated on average to be 129,783 of which 98,149 were mapped with a mean length of 431 bp. The average amount of mappable sequence data was 53 Mb. Eighty percent of these sequences overlapped the targeted region and 52% of data were mapped on target (Fig. 1).

**Figure 2:**
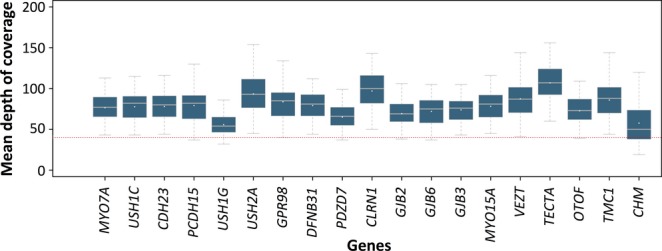
Average coverage for targeted genes. Red line represents a Depth of Coverage (DOC) of 40 reads, the defined limit for proper validation. The horizontal gray line corresponds to the median. Mean values are denoted by a white dot.

The overall depth of coverage was estimated as 77× across the whole design, ranging from 55.8× (*USH1G*) to 106.9× (*TECTA*) (Fig. 2). Among the 634 targeted regions, only 37 (5.8%) were covered less than 40× (22 regions between 20 and 40×) (Fig. S2). Eighteen of these correspond to high GC content (>60%).

### Filtering/prioritization/classification of variants

**Figure 3:**
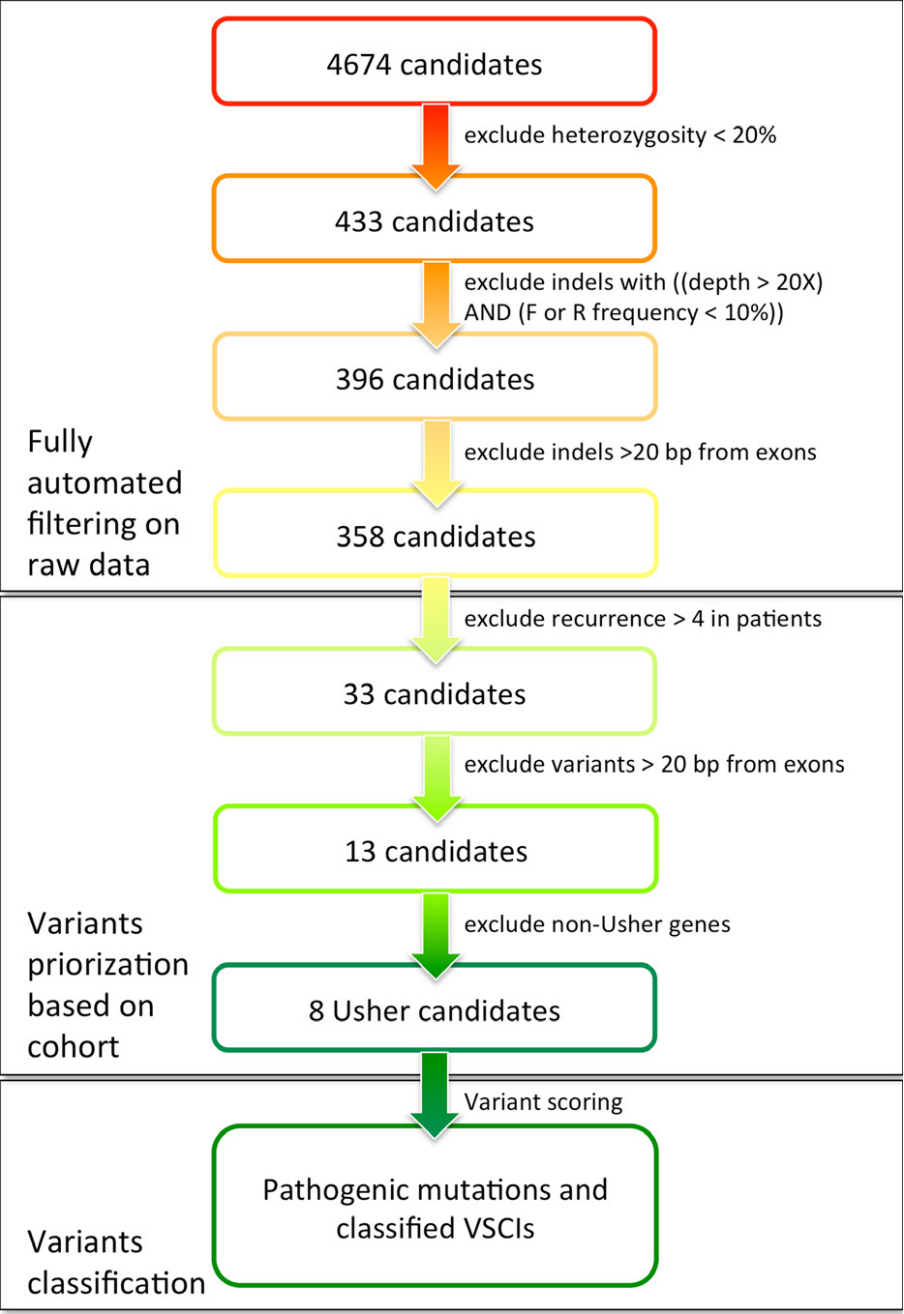
Pipeline designed for efficient filtering, prioritization, and classification of variants. Indicated figures are related to the average of the control group. F, forward strand sequences; R, reverse strand sequences; VUCS, variants of unkown clinical significance. >20 bp from exons stands for localized in introns more than 20 bp away from exon boundaries.

In the test sample, a mean of 4674 putative variants were identified per patient, however, this was reduced to eight candidate variants per patient when the analysis pipeline was applied as described above (Fig. 3). First, filtering was performed to eliminate artifacts from raw data. This task has been automated in a dedicated publicly available tool named GSdot. Then, the cohort data were used to mask likely nonpathogenic variants, that is, when variants were present in more than four patients or were more than 20 bp away from exon boundaries. The eight remaining Usher variants, representing 0.17% of the original pool of candidate variants, underwent specific analysis as detailed below.

### Sensitivity of the strategy

**Figure 4:**
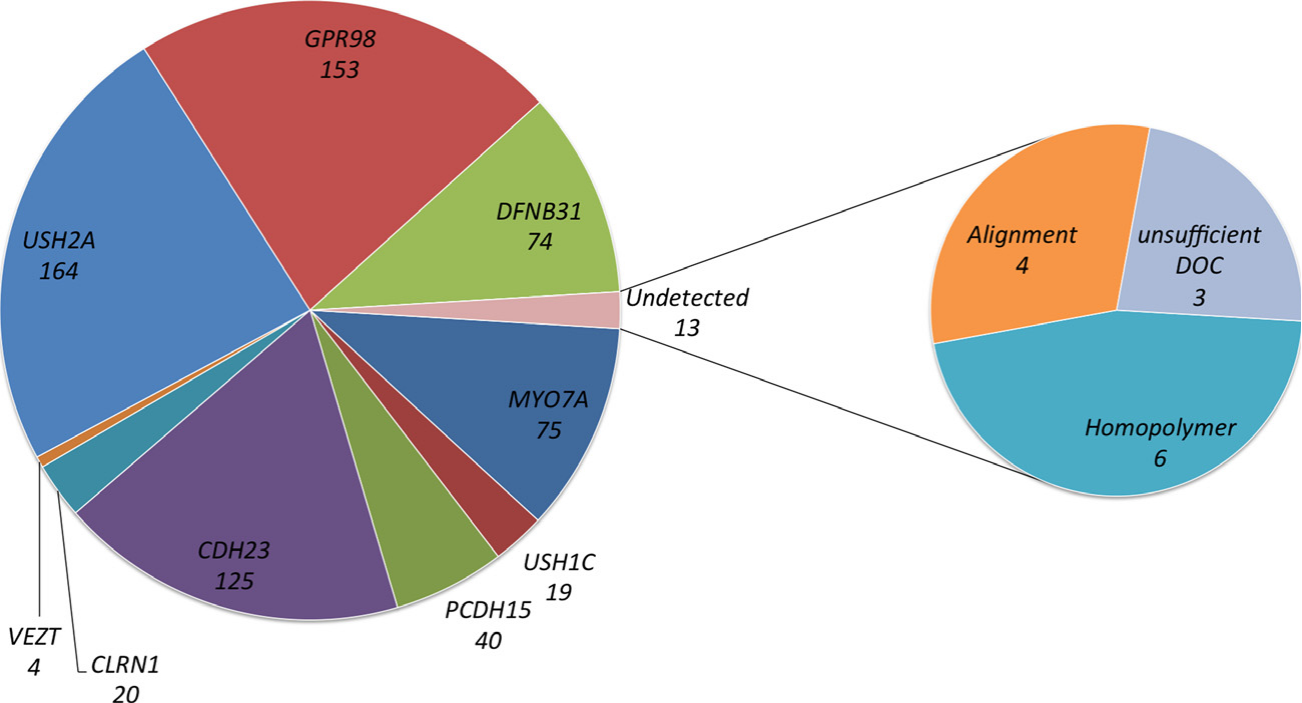
Analytical sensitivity of targeted capture approach. The graphic shows the number of variations previously identified in each gene using Sanger sequencing that were also detected by next-generation sequencing. Undetected variants are explained.

To assess the analytical sensitivity of this approach, we checked whether 687 variants (from 24 patients screened in several genes), which had been previously detected by Sanger sequencing, were also detected with NGS. These variants were widespread throughout the nine Usher genes known at the time of the study plus *VEZT* (Fig. 4). All these variations were located in exons or within the 20 bp adjacent intronic sequences, in line with the filters applied to NGS data. The detection rate by our NGS protocol was 98% (674/687). Of the 13 false-negative variants, six lay in homopolymeric regions, four could be visually detected but were misaligned and therefore not considered by the variant calling software provided by Roche, and three were localized in poorly covered regions (<40×).

### Identification of previously undetected variations in the test sample

NGS of the 47 patients of the test sample revealed more than 16,000 variants after filtering (Fig. S3). In addition to the concordant variants described above (NGS vs. Sanger), additional pathogenic mutations were detected in 12 of the patients (Table 1). In patients RP98, RP1578, U654, and RP1616, one mutation had been missed by Sanger sequencing, all in *USH2A*. A c.11864G>A mutation had not been detected in U654 and RP1616 because the sequencing primer was masking the variant, and two other mutations c.14803C>T (RP98) and c.13811+2T>G (RP1578) had not been detected because of errors in reading the Sanger sequences. In eight patients, mutations were identified in genes that had not been previously sequenced. Patients U277 and U286, clinically classified as USH2 were found to have truncating mutations in *GPR98*. Patient U1080, diagnosed as USH1 and previously found to carry a rare *MYO7A* missense, was also harboring mutations in *USH1C*. Patients RP1604 and RP1611 diagnosed as USH2, and patient RP1024 classified as USH3, were found mutated in genes (*CDH23*, *CLRN1*, and *MYO7A*, respectively) usually implicated in a different clinical subtype. Patients U996 and U585 could not be classified into any of the subtypes based on the available clinical data. Two *USH2A* mutations were identified in U996, establishing a diagnosis of USH2, but only one *MYO7A* alteration was found in U585.

**Table 1:** Mutations previously identified by Sanger sequencing and the additional mutations detected by (NGS) in 12 patients from the test sample

Patient	USH type	Gene	Mutations identified by Sanger	Additional mutations identified by NGS
U654	II	*USH2A*	c.5528C>T – p.(Pro1843Leu)	c.11864G>A – p.(Trp3955^*^)^1^
RP1616	II	*USH2A*		c.11864G>A – p.(Trp3955^*^)^1^ c.11864G>A – p.(Trp3955^*^)^1^
RP98	II	*USH2A*		c.14803C>T – p.(Arg4935^*^)^1^
RP1578	II	*USH2A*	c.2299delG – p.(Glu767fs)^1^	c.13811+2T>G – p.(?)
U996	Undef	*USH2A*	NA	c.2299delG – p.(Glu767fs)^1^ c.2176T>C – p.(Cys726Arg)
U286	II	*USH2A*	c.2299delG – p.(Glu767fs)^1^	
		*GPR98*	NA	c.10458G>A – p.(Trp3486^*^)
U277	II	*GPR98*	NA	c.13536_13537delTC – p.(Pro4513fs) c.13536_13537delTC – p.(Pro4513fs)
RP1024	III	*MYO7A*	NA	c.3610C>A – p.(Pro1204Thr) c.3764delA – p.(Lys1255fs)^1^
U585	Undef	*MYO7A*	NA	c.2283-1G>T – p.(?)^1^
U1080	I	*MYO7A*	c.5803C>A – p.(Leu1935Met)	
		*USH1C*	NA	c.311G>A – p.(Gly104Asp) c.226C>T – p.(Gln76^*^)
RP1604	II	*CDH23*	NA	c.7221C>A – p.(Tyr2407^*^) c.7221C>A – p.(Tyr2407^*^)
RP1611	II	*CLRN1*	NA	c.67G>T – p.(Gly23^*^) c.67G>T – p.(Gly23^*^)

All missense variations were classified as likely pathogenic (UV3), based on familial segregation analysis, low frequencies in public databases and in silico predictions. NA, gene not analyzed by Sanger sequencing; Undef, data not accurate enough to clearly discriminate a clinical subtype.

1 Previously described variant. References for published DNA variations as well as dbSNP identifiers are all included in USHbases.

In 14 patients carrying a single mutation identified by Sanger analysis, no additional mutations were detected by NGS.

### Usher diagnosis group

Thirteen USH patients (clinically classified as five USH1, seven USH2, and one USH3) without preliminary haplotyping or Sanger sequencing analysis underwent Usher exome screening by NGS. The previously validated filtering and prioritization strategy was applied and selected 80 USH variant candidates, an average of six variants of interest per patient. Among those, some have already been described as nonpathogenic in our local database or in USHbases and were eliminated. The remaining variants are shown in Table 2. We then applied our multistep analysis to classify these 49 variants (Roux et al. 2011).

**Table 2:** Classification of 49 variations identified in the Usher group

Patient	USH type	Gene	Variant	Class	Main contributor
U1067	I	*MYO15A*	c.10242C>T – p.(=)	UV1	b, e
U1084	I	*MYO7A*	c.6220C>T – p.(Pro2074Ser)	UV2	d
		*USH1C*	c.307C>T – p.(Arg103Cys)	UV3	d
		*USH2A*	c.14662A>T – p.(Thr4888Ser)	UV1	d
		*GPR98*	c.2727C>A – p.(=)	UV2	e
			c.9366A>G – p.(=)	UV2	e
		*PDZD7*	c.1916C>G – p.(Ala639Gly)	UV1	d
U1093	II	*USH1C*	c.2585C>T – p.(Pro862Leu)	UV1	d
			c.101A>G – p.(His34Arg)	UV2	b
		*GPR98*	c.8992_8994delinsAAGTTCC – p.(Ala2998fs)	Pathogenic	a
			c.8992_8994delinsAAGTTCC – p.(Ala2998fs)		
		*MYO15A*	c.7468G>A – p.(Ala2490Thr)	UV1	b
U1120	I	*CDH23*	c.9904G>A – p.(Glu3302Lys)	UV3	d
U1141	II	*USH2A*	c.1055C>T – p.(Thr352Ile)	UV4	c, d^2^
			c.949C>A – p.(?)	UV4	c^2^
U1148	II	*USH2A*	c.14475_14484del – p.(Ala4827fs)	Pathogenic	g, a
			c.14766delG – p.(Trp4922^*^)	Pathogenic	g, a
		*PDZD7*	c.2144C>T – p.(Pro715Leu)	UV1	b
		*MYO15A*	c.54G>A – p.(=)	UV1	b, e
			c.7908C>T – p.(=)	UV1	b, e
U1157	I	*CDH23*	c.9278+5G>C – p.(?)	UV4	f
			c.9278+5G>C – p.(?)		
U1163	I	*CDH23*	c.5015_5016delAT – p.(Tyr1672fs)	Pathogenic	a
			c.6829+1G>A – p.(?)	UV4	e
		*PDZD7*	c.1011C>T – p.(=)	Neutral	b, e
		*MYO15A*	c.1385G>A – p.(Gly462Asp)	UV1	b
			c.5133+15A>G – p.(=)	UV1	b, e
U1167	II	*USH2A*	c.1256G>T – p.(Cys419Phe)	UV4	c, d^2^
			c.2882delA – p.(His961fs)	Pathogenic	a
		*GPR98*	c.10820T>C – p.(Val3607Ala)	UV3	d
		*CDH23*	c.4891G>A – p.(Ala1631Thr)	UV1	d
U1178	II	*GPR98*	c.2864C>A – p.(Ser955^*^)	Pathogenic	a
			c.2864C>A – p.(Ser955^*^)		
			c.17756-4A>G – p.(=)	UV2	e
			c.17756-4A>G – p.(=)		
U1170	I	*MYO7A*	c.472G>A – p.(Gly158Arg)	UV3	d
			c.5502G>A – p.(Trp1834^*^)	Pathogenic	a
		*CDH23*	c.9319G>T – p.(Gly3107Trp)	UV2	h^1^
		*CLRN1*	c.472+4C>T – p.(=)	Neutral	b
		*VEZT*	c.1396G>A – p.(Glu466Lys)	UV2	h
		*MYO15A*	c.3413A>G – p.(Gln1138Arg)	Neutral	b
			c.4655+11G>A – p.(=)	UV1	b
U1171	II	*GPR98*	c.7129C>T – p.(Arg2377^*^)	Pathogenic	a
			c.13536_13537delTC – p.(Pro4513fs)	Pathogenic	a
		*MYO7A*	c.1134C>T – p.(=)	UV2	e
U1185	II	*USH2A*	c.653T>A – p.(Val218Glu)	UV3	c, d^2^
			c.13010C>T – p.(Thr4337Met)	UV3	d^2^
		*DFNB31*	c.1204-17A>G – p.(=)	UV2	e
		*USH1C*	c.496+1C>T – p.(?)	Pathogenic	a^2^
		*VEZT*	c.1831+4A>G – p.(=)	UV2	e^1^
		*MYO15A*	c.9478C>T – p.(Leu3160Phe)	UV2	b, d
			c.10182G>A – p.(=)	UV1	b, e

Contributors to classification: a, protein translation predicts a PTC; b, allele frequency (public databases or control samples analyzed by our laboratory); c, allele frequency (patients); d, in silico predictions (missense variants); e, in silico predictions (splicing); f, minigene analysis; g, segregation analysis; h, patient genotype.

1 Variant that could alter splicing.

2 Previously described variant. References for published DNA variations as well as dbSNP identifiers are all included in USHbases.

**Figure 5:**
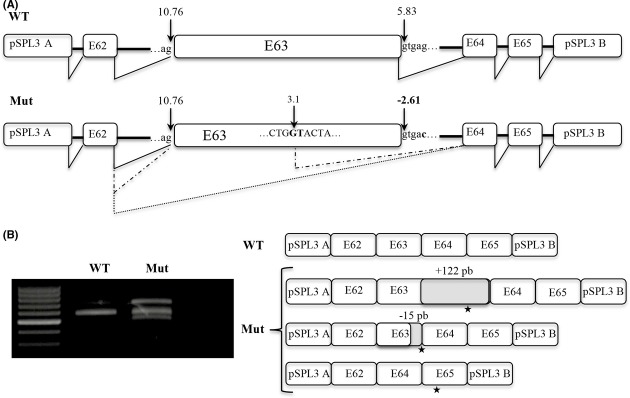
Ex vivo analysis of *CDH23* c.9278+5G>C. (A) Schematic wild-type (WT) and mutated (Mut) pSPL3 minigenes including exons 62 to 65 of *CDH23*. Exons are represented by boxes and introns by horizontal lines. The strength of splice sites is estimated by the MaxEnt scores. (B) Agarose gel and schematic sequences of transcripts obtained by RT-PCR from wild-type (WT) and mutated (Mut) minigenes. Black stars indicate premature termination codons (PTC).

NGS successfully identified the pathogenic genotype in 10 out of 13 patients (77%). Patients U1157 and U1163 were found to have *CDH23* alterations. U1157 carried a newly described variant in position +5 of exon 63, and a minigene analysis was performed to assess the impact of the substitution on the splicing process. The c.9278+5G>C variant leads to a premature stop codon either by a retention of intron 63, a deletion of the last fifteen nucleotides of exon 63 (use of a cryptic donor splice site) or a total skipping of this exon (Fig. 5). USH1 patient U1170 carried mutations in *MYO7A*, a truncating mutation and a newly described missense, p.(Gly158Arg). Among the three patients carrying pathogenic mutations in *GPR98*, two (U1093 and U1178) were homozygotes for truncating mutations and the other (U1171) was compound heterozygous for two truncating mutations. Four patients (U1141, U1148, U1167, U1185) were *USH2A* compound heterozygotes; three truncating mutations (p.(Ala4827fs), p.(Trp4922*), p.(His961fs)) are newly described whereas the other five are well known (see USHbases): four missense (p.(Thr352Ile), p.(Cys419Phe), p.(Val218Glu), p.(Thr4337Met)) and a splicing alteration (c.949C>A).

In two additional patients, NGS detected a single rare candidate UV3 variant, *USH1C* p.(Arg103Cys) in U1084 and *CDH23* p.(Glu3302Lys) in U1120. All *USH1C* and *CDH23* exons were further sequenced by Sanger in U1084 and in U1120, respectively, to avoid any missed mutations in a homopolymeric region.

Finally, in only one patient, U1067 clinically diagnosed as USH3, no candidate pathogenic alteration could be identified.

In addition to the pathogenic and UV3 variants identified, several rare variants were detected among the 13 patients. Most of them were classified as nonpathogenic. Interestingly, U1185 carried, in addition to an *USH2A* pathogenic genotype, the c.496+1G>T variant in *USH1C*.

### NSHL diagnosis group

We assigned unambiguous disease-causing mutations in only 1/11 cases (S91) although a number of potentially disease-causing changes were present as heterozygotes in a number of cases (Table 3). S91, with Spanish and Algerian origins, carried a homozygous mutation p.(Arg389*) in *TMC1*. *TMC1* is the sixth most common cause of recessive HL worldwide (Hilgert et al. 2009), and the most prevalent in Iran, Turkey, Israel, and Jews of Moroccan origin (Brownstein et al. 2011).

**Table 3:** List of candidate variants detected in NSHL group

Patient	Documented consanguinity	Gene	Variant	Class	Main contributor
S4	Yes	*GJB3*	c.94C>T – p.(Arg32Trp)	UV1	b^1^
		*PCDH15*	c.1205G>C – p.(Gly402Ala)	UV2	d
		*USH2A*	c.8575C>T – p.(Arg2859Cys)	UV1	d
S11	Yes	*–*	–	–	–
S25	Yes	*MYO7A*	c.849+7C>G – p.(=)	UV2	e
S28	Yes	*PCDH15*	c.875C>G – p.(Pro292Arg)	UV3	b, d, h
			c.55T>A – p.(Ser19Thr)	UV1	d, h
S91	Yes	*TMC1*	c.1165C>T – p.(Arg389^*^)	Pathogenic	a^1^
			c.1165C>T – p.(Arg389^*^)		
S634	Yes	*USH2A*	c.14904C>T – p.(=)	UV2	e
S789	Yes	*MYO7A*	c.687C>T – p.(=)	UV3	e
S334	No	*CDH23*	c.6050-9G>A – p.(?)	Pathogenic	c^1^
S385	No	*MYO15A*	c.1656C>T – p.(=)	UV2	e
		*OTOF*	c.1966C>T – p.(Arg656Trp)	UV1	d
S565	No	*GJB2*	c.35delG – p.(Gly12 fs)	Pathogenic	a^1^
		*TECTA*	c.3239A>T – p.(Asp1080Val)	UV2	d
			c.3456G>A – p.(=)	UV2	e
		*MYO15A*	c.806C>T – p.(Pro269Leu)	UV1	d
		*PDZD7*	c.1452G>T – p.(=)	UV2	e
S660	No	*OTOF*	c.1392+1G>T – p.(?)	Pathogenic	a
		*CDH23*	c.3995T>G – p.(Ile1332Ser)	UV3	d

Contributors to classification: a, protein translation predicts a PTC; b, allele frequency (public databases or control samples analyzed by our laboratory); c, allele frequency (patients); d, in silico predictions (missense variants); e, in silico predictions (splicing); f, minigene analysis; g, segregation analysis; h, patient genotype. No candidate pathogenic alterations have been identified for patient S11.

1 Previously described variant. References for published DNA variations as well as dbSNP identifiers are all included in USHbases.

Gene and protein reference sequences are listed in Table S2 of the online Supporting Information.

## Discussion

### NGS in clinical services

As a reference laboratory, we have developed over the last 6 years a comprehensive approach that allows a mutation detection rate of more than 90% of cases for USH1 (Roux et al. 2006, 2011) and USH2 (Baux et al. 2007; Besnard et al. 2012). This includes screening for large rearrangements (Le Guédard et al. 2007; Roux et al. 2011) and the analysis of USH transcripts from nasal epithelial cells (Vaché et al. 2010, 2012) as well as the development of a multistep analysis to interpret the variants of unknown clinical significance (Baux et al. 2013). Because Sanger sequencing is time consuming, we included a preliminary linkage analysis at the USH1 loci that prioritizes the gene to be sequenced in 44% of the cases. In our cohort, *MYO7A* is the most prevalent gene responsible for more than 60% of the USH1 cases. *USH2A* accounts for 80% of Usher type 2 cases (Besnard et al. 2012) and is usually screened as a first step unless siblings are available or consanguinity is present, in which case, haplotype analysis is performed. In the present study, we have evaluated sequencing of the targeted Usher exome coupled with a benchtop NGS machine. Sequencing in parallel all candidate genes has clear advantages as it can resolve not only atypical USH cases but also cases with a misclassified or poorly defined clinical subtype. We found that despite a sensitivity of 98%, failure to identify pathogenic mutations, particularly one of the founder mutations, was real and was inherent to limitations in the technology, particularly the difficulty of sequencing short runs of repeats. Taking all this into account, we have worked out a “decision-making diagram” as represented on Figure S4. If clinical criteria are clearly indicative of USH1, 2, or 3, we would still recommend performing haplotype analysis and/or Sanger sequencing of the two major genes (i.e., *MYO7A* for USH1 and *USH2A* for USH2); if negative, then NGS should be performed. For any atypical/undefined cases, NGS should be performed first.

### Raw data quality and technical issues

Using the Roche GS junior 454 sequencer, we were able to generate an average of 40 Mb of pertinent nucleotides per run (Fig. 1). The deficit of 10 Mb per run due to off-target sequences is a feature of the sequence capture method and is inevitable in order to obtain a reasonable coverage for the regions of interest. While we observed that 96% of our target regions were covered by at least 40 reads (Fig. 2 and Fig. S2), and that 98% of variants previously identified by Sanger sequencing were correctly found by NGS (Fig. 4), we identified some weaknesses in the base-calling and alignment system used (namely 454 base caller and GS Reference Mapper which are provided by Roche). Misalignment was noted not only in homopolymeric regions but also in some neighboring regions (e.g., the *USH2A* c.2299delG mutation lies in the vicinity of a stretch of 6 A and could not be detected at first when using the default parameters, see below). This is very important as such homopolymers are frequent causes of mutation due to slippage. Base calling and alignment, two crucial steps, must be improved in the future. Base calling is defined as the analysis of the sensor data to predict the individual bases (Ledergerber and Dessimoz 2011). In the case of 454 pyrosequencing, this relies on the quantification of emitted light during a single nucleotide flow (Margulies et al. 2005). Until recently, only two base callers were available to analyze 454 data (Datta et al. 2010), the native 454 base caller and Pyrobayes (Quinlan et al. 2008). Pyrobayes has been reported to be more accurate than the built-in 454 base caller, for example, in substitution error rate, but it does not improve errors due to homopolymeric regions. To address this point, a new method called HPcal has been claimed to reduce homopolymeric length errors by 35% (Beuf et al. 2012).

The quality of the generated data could also be improved by modifying the alignment method. Alignment of hundreds of thousands of reads on a reference genome is a huge task, and therefore dedicated software needs to proceed in two steps: a fast mapping of the reads is first performed on the genome to identify candidate regions and is followed by a fine alignment of the same reads on those regions. The latter is realized using a classical algorithm such as Smith–Watermann (1981), but the first mapping on candidates regions is achieved by different methods. Several software packages are available. The most efficient ones, such as BWA (Li and Durbin 2009) or Bowtie (Langmead et al. 2009), are based on the Burrows–Wheeler transform approach. One of the advantages of 454 sequencing is to generate long reads (average of 431 bp in this study), but this reduces the number of optimized alignment and base-calling methods that are available. Among those, AGILE (AliGnIng Long rEads) seems to be promising in terms of accuracy, memory usage, and speed (Misra et al. 2011).

### Filtering, prioritization, and classification of variants

We divided the filtering, prioritization, and classification of our NGS data into three distinct stages (Fig. 3). Analysis of the copious amounts of data generated raises problems at two levels, firstly in checking the validity of the reads (steps 1–3) and, secondly, in determining relevance to disease-causing changes. All the settings were done on the test sample. The first step aims to eliminate the maximum number of false positives without removing the true positives from the dataset. This step has been automated with GSdot software. The generic software GS Reference Mapper generates two lists of possible DNA variations, one containing all signals, and a second supposedly including only the true variants. We first followed the recommendations of the manufacturer and worked with the “cleaned file” (High Confidence Variations file) but realized that the most frequent mutation in *USH2A* c.2299delG, responsible for 10–45% of *USH2A* pathogenic alleles in Europe (Dreyer et al. 2008; Aller et al. 2010; Le Quesne Stabej et al. 2012), was systematically excluded from the second list. This pinpoints a drawback of the software, which is poor in detecting changes within or near homopolymeric stretches. We therefore chose to use as input for our custom software GSdot the complete list of aligned DNA alterations, which explains the relatively high number of candidate DNA variants at the very beginning of the workflow (mean 4674), the majority of which, 92.3% (i.e., 4316/4674) are removed/excluded by the first filter.

The second stage consisted in the selection of candidate pathogenic variants. This step remains to be automated. We chose not to depend on external software such as pathogenic predictors or external databases. This strategy has proven to be effective as only eight variants per patient remained after this screening. At this point, every variant was confirmed by Sanger sequencing, which revealed that a few false positives were still present, and this suggests that more stringent filters need to be applied. External software and databases were fully integrated in the third step, which focused on the classification of the Usher candidate variants. Our multistep strategy of classification was applied to any putative splicing alteration as well as to variants expected to impact on the protein structure, and proved to be very efficient (Roux et al. 2011; Baux et al. 2013). This was possible thanks to our in-house experience of molecular alterations of the Usher genes in USH patients, our creation and maintenance of USMA and of an internal database, and curation of USHbases which contains and correlates all the published information.

The massive amounts of data generated by NGS represent both a challenge for analysis and a powerful tool to define sequence variations. The number of variants identified in 19 genes by Sanger sequencing (2475) or NGS (16,851, after automated filtering) in the 47 patients of the test sample are displayed in Figure S3. These data provide a unique resource in terms of distribution of variants identified in patients which will greatly facilitate future diagnoses.

Data from the test sample illustrate the limitations of Sanger sequencing in terms of effectiveness in identifying pathogenic genotypes in the following situations: (i) high genetic heterogeneity, particularly if the most prevalent genes (i.e., *MYO7A* and *USH2A*) are not involved; (ii) large genes to be sequenced; and (iii) patients with atypical or poorly characterized USH. In these cases, Sanger sequencing is time consuming and expensive which limits the completeness of service in routine diagnostics labs. We have indeed improved USH diagnosis for 12/47 patients mainly because all relevant genes were examined exhaustively.

### Sensitivity of the strategy

We have found the NGS approach sensitive (with a rate of 98%) and, apart from the false negatives within homopolymer stretches already discussed, the remainder were three lying in poorly covered regions within the *DFNB31* region, which requires optimization of the design. In reality, we have particularly validated the high capacity of the 454 sequencing to identify nucleotide substitutions (670 among the 674 identified variations), which account for most of the variants, pathogenic or not, in these genes. Until methods for detecting changes within homopolymers are improved, Sanger sequencing should be performed systematically for patients in whom one pathogenic variant is identified by NGS.

No causative mutation could be found in 19 patients in any candidate USH genes or in the NSHL genes also included in the design. Deep intronic mutations (such as c.7595-2144A>G recently identified in *USH2A* by mRNA studies [Vaché et al. 2012]) may be involved in rare cases. Most likely explanation for negative cases is that the patients are not “classical” USH patients, that is, expected to carry mutations in “USH genes”. Only Whole-Exome Sequencing (WES) or Whole-Genome Sequencing (WGS) are likely to bring answers and may redefine the clinical diagnosis for some cases.

Among 19 patients carrying a single mutation, 10 were heterozygous for an *USH2A* pathogenic allele. The carrier frequency of an *USH2A* mutation is estimated to be 1/70 in U.K. (95% CI = 1/333–1/40) (Le Quesne Stabej et al. 2012) and in our cohort (95% CI = 1/111–1/53) (A. F. Roux, unpublished results), therefore these patients cannot all be random carriers and it is most likely that the second mutation has not yet been detected. For two patients, U838 carrying *MYO7A* p.(Cys31*), a common mutation in Scandinavian populations (Janecke et al. 1999), and U585 carrying c.2283-1G>T, a common mutation in North African populations (see USHbases), the clinical description did not allow classification into a particular subgroup so that the question remains open as to whether they are random carriers.

Among the genotyped USH patients, two could be put forward as potential oligogenic or digenic cases. Patient U1185 (presenting with typical USH2 clinical signs) carries three pathogenic mutations: two in *USH2A* and the *USH1C* c.496+1G>T splice mutation. Patient U286 carries the *USH2A* c.2299delG and the *GPR98* p.(Trp3486*) truncating mutation. Although digenic mechanism could be postulated, this patient could just as well be a random carrier of the frequent c.2299delG mutation with two pathogenic *GPR98* mutations. We have already described a patient as a random c.2299delG carrier associated with a *CDH23* linked USH1 syndrome (Roux et al. 2011).

Incidental findings are a direct consequence of exhaustive screening with NGS. Although the number of genes screened with this approach is targeted, it already pinpoints the presence of additional mutations, which probably just reflect the carrier rate frequency in the general population. In USH, the carrier frequency of one USH gene mutation could be estimated as 1/42 (95% CI = 1/90–1/27), considering a MAF for c.2299delG of 0.009 (EVS and [Baux et al. 2007]) with this mutation representing 7.9% of the pathogenic USH alleles in our series.

This pilot study of NGS applied to the molecular diagnosis of an heterogeneous disorder emphasizes the need of special expertise of the genes analyzed for correct interpretation of variants in a clinical context. In-house databases cumulating patients' data, as well as public available databases, will be of great help to develop efficient diagnosis.

## Supporting Information

Figure S1 Patients included in the study.

Figure S2 Box-plots of average DOC for targeted regions. Detailed coverage for the 634 regions is shown gene by gene. Boxes are showed in red when mean DOC in the region is lower than 409. Red line represents a DOC of 40 reads, the minimum limit for a proper validation. The horizontal gray line corresponds to the median. The mean value is marked with a white dot.

Figure S3 Comparison between useful data generated by NGS and Sanger sequencing in Usher test sample. Numbers in the sphere correspond to the sum of variants identified in each approach.

Figure S4 Inclusion of NGS in the decision-making diagram for molecular diagnosis of USH patients.

Table S1 List of 47 Usher patients included in test sample. The genes previously studied using Sanger sequencing or aCGH for each subject are marked with a cross and the identified putative mutations are displayed (all the mutations were detected in the heterozygous state).

Table S2 Table of 19 human genes targeted for the nextgeneration sequencing. Additional exon(s) refers to the number of exons differing from the main isoform.
