# Supplementary figures and images for: Experience of targeted Usher exome sequencing as a clinical test
Source: Mol Genet Genomic Med. 2013 Jul 10;2(1):30–43. doi: 10.1002/mgg3.25 (PMC3907913; doi:10.1002/mgg3.25)

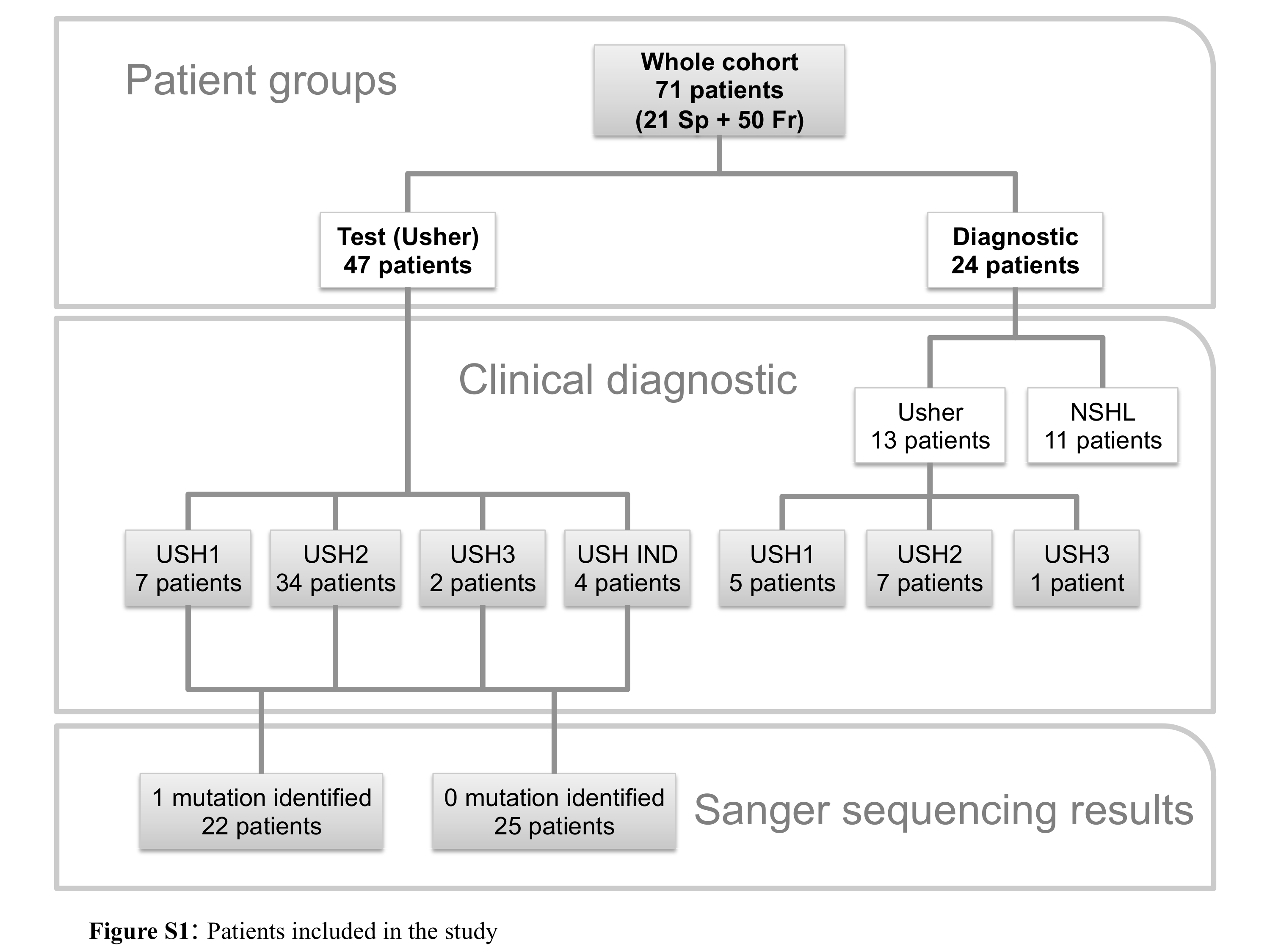

Supplement: Figure S1 — Patients included in the study. [file mgg30002-0030-sd1.tif]

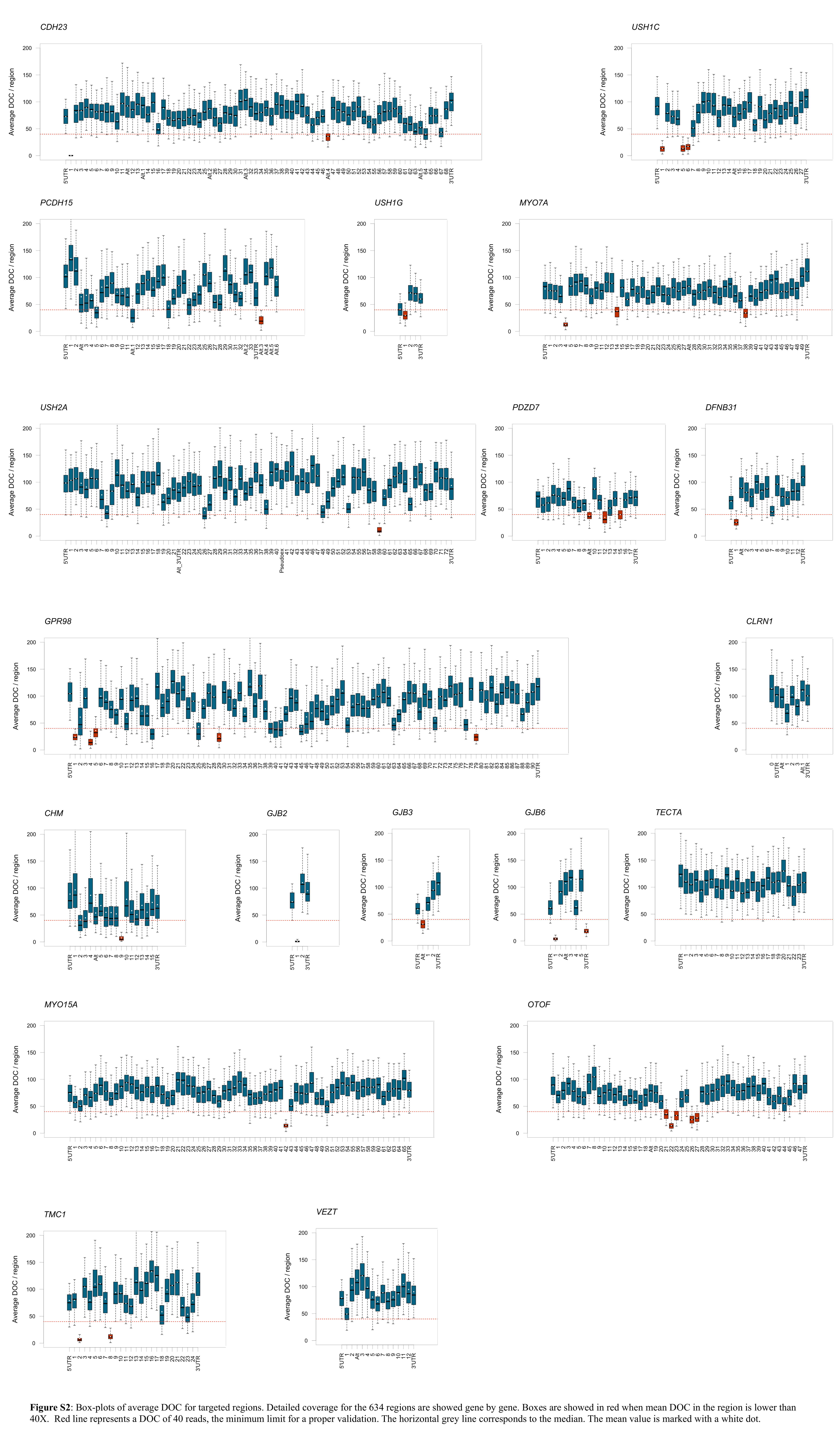

Supplement: Figure S2 — Box-plots of average DOC for targeted regions. Detailed coverage for the 634 regions is shown gene by gene. Boxes are showed in red when mean DOC in the region is lower than 409. Red line represents a DOC of 40 reads, the minimum limit for a proper validation. The horizontal gray line corresponds to the median. The mean value is marked with a white dot. [file mgg30002-0030-sd2.tif]

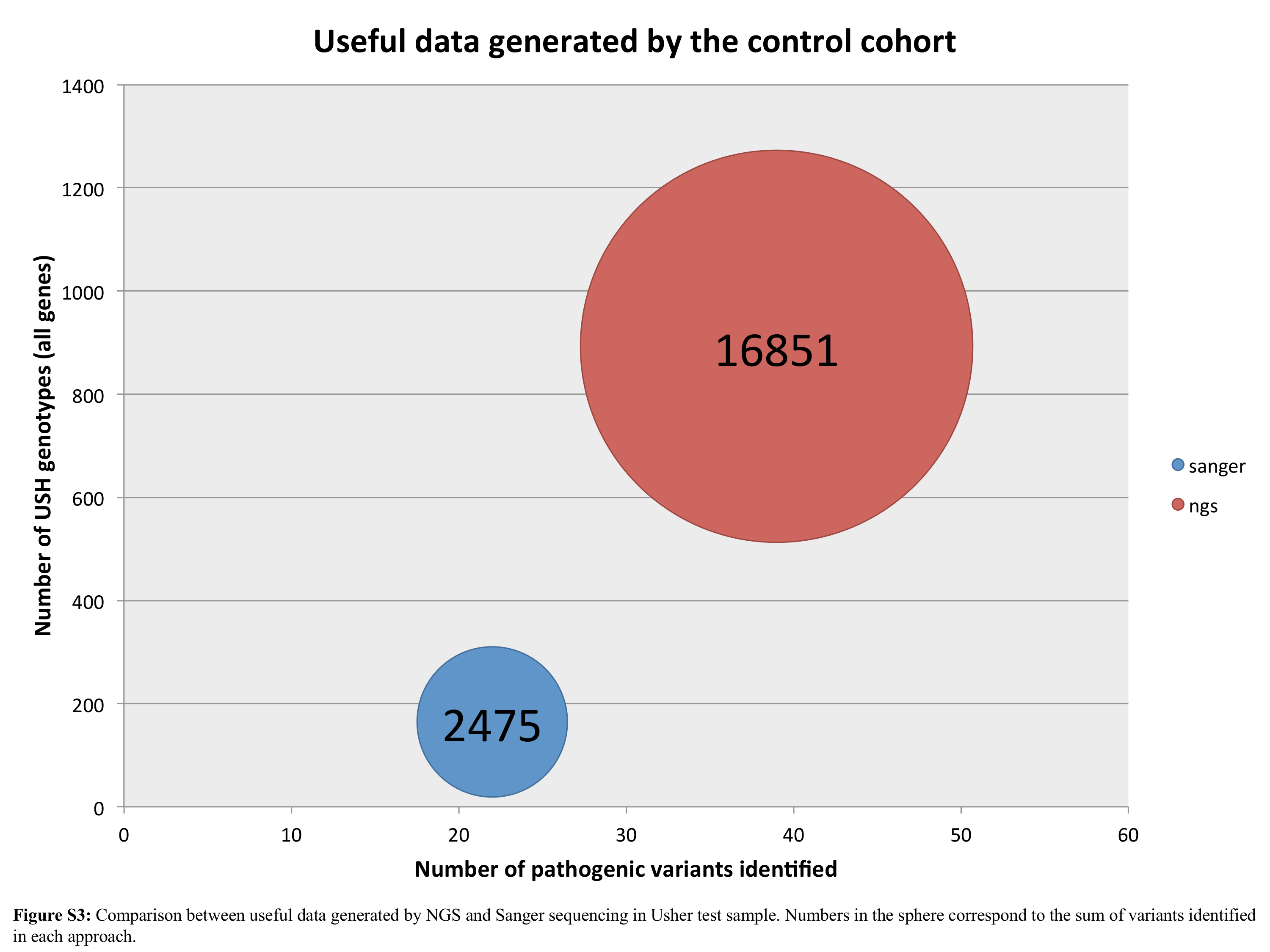

Supplement: Figure S3 — Comparison between useful data generated by NGS and Sanger sequencing in Usher test sample. Numbers in the sphere correspond to the sum of variants identified in each approach. [file mgg30002-0030-sd3.tif]

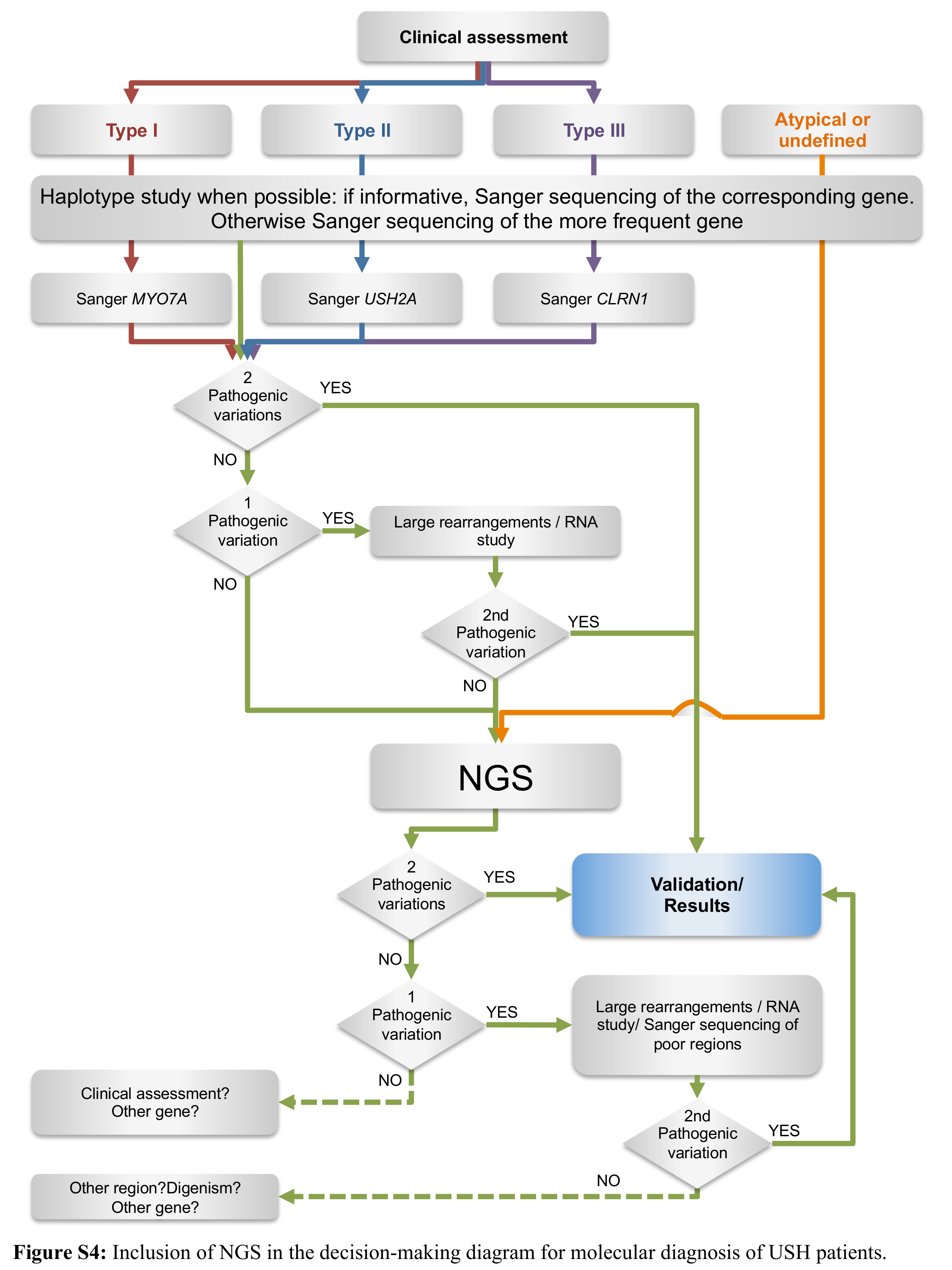

Supplement: Figure S4 — Inclusion of NGS in the decision-making diagram for molecular diagnosis of USH patients. [file mgg30002-0030-sd4.tif]
