# Supplementary material for: Experience of targeted Usher exome sequencing as a clinical test
Source: Mol Genet Genomic Med. 2013 Jul 10;2(1):30–43. doi: 10.1002/mgg3.25 (PMC3907913; doi:10.1002/mgg3.25)
Supplement: Table S1 — List of 47 Usher patients included in test sample. The genes previously studied using Sanger sequencing or aCGH for each subject are marked with a cross and the identified putative mutations are displayed (all the mutations were detected in the heterozygous state). [file mgg30002-0030-sd5.docx]

| **PATIENT** | **USH Type** | ***MYO7A*** | ***USH1C*** | ***CDH23*** | ***PCDH15*** | ***USH1G*** | ***USH2A*** | ***GPR98*** | ***DFNB31*** | ***PDZD7*** | ***CLRN1*** | **Results before NGS** | **aCGH** |
| --- | --- | --- | --- | --- | --- | --- | --- | --- | --- | --- | --- | --- | --- |
| U810 | I | X | X | X | X |  |  |  |  |  |  | NM_033056.3(*PCDH15*):c.1441-1G>A | X |
| U331 | I | X | X | X | X | X |  |  | X | X | X | NM_033056.3(*PCDH15*):del E18-26 | X |
| U1080 | I | X |  |  |  |  |  |  |  |  |  | NM_000260.3(*MYO7A*):p.(Leu1935Met) |  |
| U321 | I | X |  | X | X |  | X |  |  |  | X | - |  |
| U503 | I |  |  | X | X |  |  |  |  |  | X | - |  |
| U584 | I | X | X | X | X |  |  |  |  |  | X | - |  |
| U740 | I | X | X | X | X |  | X |  |  |  | X | - |  |
| U283 | II |  |  |  |  |  | X |  |  | X |  | NM_206933.2(*USH2A*):p.(Gly2039fs) |  |
| U466 | II | X | X | X | X |  | X | X |  | X | X | NM_206933.2(*USH2A*):p.(Cys759Phe) | X |
| U286 | II |  |  |  |  |  | X |  |  |  | X | NM_206933.2(*USH2A*):p.(Glu767fs) |  |
| RP1360 | II |  |  |  |  |  | X |  |  |  |  | NM_206933.2(*USH2A*):p.(Cys759Phe) | X |
| RP1485 | II |  |  |  |  |  | X |  |  |  |  | NM_206933.2(*USH2A*):p.(Glu404fs) | X |
| RP1578 | II |  |  |  |  |  | X |  |  |  |  | NM_206933.2(*USH2A*):p.(Glu767fs) | X |
| RP1617 | II |  |  |  |  |  | X |  |  |  |  | NM_206933.2(*USH2A*):p.(His308fs) | X |
| RP1635 | II |  |  |  |  |  | X |  |  |  |  | NM_206933.2(*USH2A*):p.(Cys759Phe) | X |
| RP963 | II |  |  |  |  |  | X |  |  |  |  | NM_206933.2(*USH2A*):p.(Cys759Phe) | X |
| U329 | II |  |  |  |  |  | X |  | X |  | X | NM_001195263.1(*PDZD7*):p.(Arg56fs) |  |
| U787 | II |  |  |  |  |  | X | X | X | X |  | NM_032119.3(*GPR98*):p.(Arg5688*) | X |
| RP1634 | II |  |  |  |  |  | X | X | X |  |  | NM_032119.3(*GPR98*):p.(Gln5796*) | X |
| U496 | II |  |  | X |  |  | X | X | X | X | X | NM_206933.2(*USH2A*):p.(Arg1777Trp) | X |
| U654 | II |  |  |  |  |  | X | X | X | X | X | NM_206933.2(*USH2A*):p.(Pro1843Leu) | X |
| U932 | II |  |  |  |  |  | X |  |  |  |  | NM_206933.2(*USH2A*):p.(Cys717Gly) | X |
| RP1600 | II |  |  |  |  |  | X | X | X |  |  | NM_206933.2(*USH2A*):p.(Val382Met) |  |
| U391 | II |  |  |  |  |  | X | X | X | X | X | NM_032119.3(*GPR98*):p.(Gly2045Arg) | X |
| U444 | II |  | X | X | X |  |  |  | X |  | X | - | X |
| U583 | II |  |  |  |  |  | X | X | X | X | X | - |  |
| U670 | II |  |  |  |  |  | X | X | X | X | X | - |  |
| U767 | II | X |  | X |  |  | X | X | X | X | X | - |  |
| U436 | II |  |  |  |  |  | X | X | X | X | X | - |  |
| U277 | II |  |  |  |  |  | X |  | X | X | X | - |  |
| RP98 | II |  |  |  |  |  | X |  |  |  |  | - |  |
| RP1059 | II |  |  |  |  |  | X | X | X |  |  | - |  |
| RP1278 | II |  |  |  |  |  | X | X | X |  |  | - |  |
| RP1417 | II |  |  |  |  |  | X |  | X |  |  | - |  |
| RP1448 | II |  |  |  |  |  | X | X | X |  |  | - |  |
| RP1604 | II |  |  |  |  |  | X | X | X |  |  | - |  |
| RP1606 | II |  |  |  |  |  | X | X | X |  |  | - |  |
| RP1611 | II |  |  |  |  |  | X | X | X |  |  | - |  |
| RP1616 | II |  |  |  |  |  | X | X | X |  |  | - |  |
| RP1636 | II |  |  |  |  |  | X | X | X |  |  | - |  |
| RP659 | II |  |  |  |  |  | X |  |  |  |  | - |  |
| RP1024 | III |  |  |  |  |  | X | X | X |  |  | - |  |
| RP1612 | III |  |  |  |  |  | X | X | X |  |  | - |  |
| U838 | Undef | X |  | X |  |  | X | X | X | X | X | NM_000260.3(*MYO7A*):p.(Cys31*) | X |
| U461 | Undef | X | X | X | X |  | X |  | X |  | X | NM_022124.5(*CDH23*):p.(Asp1130Tyr) | X |
| U585 | Undef |  |  |  |  |  | X | X | X | X | X | - |  |
| U996 | Undef |  |  |  |  |  |  |  |  |  | X | - |  |
|  |  |  |  |  |  |  |  |  |  |  |  |  |  |
| **Table S1:** List of 47 Usher patients included in test sample. The genes previously studied using Sanger sequencing or aCGH for each subject are marked with a cross and the identified putative mutations are displayed (all the mutations were detected in heterozygous state). | | | | | | | | | | | | | |
