# Supplementary material for: Experience of targeted Usher exome sequencing as a clinical test
Source: Mol Genet Genomic Med. 2013 Jul 10;2(1):30–43. doi: 10.1002/mgg3.25 (PMC3907913; doi:10.1002/mgg3.25)
Supplement: Table S2 — Table of 19 human genes targeted for the nextgeneration sequencing. Additional exon(s) refers to the number of exons differing from the main isoform. [file mgg30002-0030-sd6.docx]

| **Chr** | **gene** | **RefSeq AccNo (nucleotide)** | **RefSeq AccNo (protein)** | **exons** | **size (bp)** | **coding size (bp)** | **Amino acids** | **Alternative RefSeq (nucleotide)** | **Alternative RefSeq (protein)** | **Additional exon(s)** | **size (bp)** |
| --- | --- | --- | --- | --- | --- | --- | --- | --- | --- | --- | --- |
| 1 | *GJB3* | NM_001005752.1 variant 2 | NP_001005752.1 isoform a | 2 | 1777 | 813 | 270 | NM_024009.2 variant 1 | NP_076872.1 isoform a | 1 | 591 |
| 1 | *USH2A* | NM_206933.2 variant 2 | NP_996816.2 isoform b | 72 | 18883 | 15609 | 5202 | NM_007123.5 variant 1 | NP_009054.5 isoform a | 1 | 1534 |
| 2 | *OTOF* | NM_194248.2 variant 1 | NP_919224.1 isoform a | 47 | 7171 | 5994 | 1997 | NM_194322.2 variant 3 | NP_919303.1 isoform c | 1 | 395 |
| 3 | *CLRN1* | NM_001195794.1 variant 5 | NP_001182723.1 isoform d | 4 | 2398 | 738 | 245 | NM_052995.2 variant 4 | NP_443721.1 isoform c | 2 | 1129 |
| 5 | *GPR98* | NM_032119.3 variant 1 | NP_115495.3 isoform 1 | 90 | 19333 | 18921 | 6307 |  |  |  |  |
| 10 | *PCDH15* | NM_033056.3 variant C | NP_149045.3 isoform CD1-4 | 33 | 7021 | 5868 | 1955 | NM_001142771.1 variant K | NP_001136243.1 isoform CD3-1 | 1 | 4359 |
|  |  |  |  |  |  |  |  | NM_001142769.1 variant I | NP_001136241.1 isoform CD2-1 | 3 | 990 |
|  |  |  |  |  |  |  |  | NM_001142770.1 variant J | NP_001136242.1 isoform CD2-2 | 1 | 74 |
|  |  |  |  |  |  |  |  | NM_001142773.1 variant H | NP_001136245.1 isoform CD1-10 | 1 | 6 |
|  |  |  |  |  |  |  |  | NM_001142763.1 variant A | NP_001136235.1 isoform CD1-1 | 1 | 16 |
| 10 | *CDH23* | NM_022124.5 variant 1 | NP_071407.4 isoform 1 | 69 | 11134 | 10065 | 3354 | NM_001171932.1 variant 5 | NP_001165403.1 isoform 5 | 1 | 402 |
|  |  |  |  |  |  |  |  | NM_052836.3 variant 2 | NP_443068.1 isoform 2 | 1 | 1005 |
|  |  |  |  |  |  |  |  | NM_001171931.1 variant 4 | NP_001165402.1 isoform 4 | 1 | 612 |
|  |  |  |  |  |  |  |  | NM_001171930.1 variant 3 | NP_001165401.1 isoform 3 | 1 | 739 |
|  |  |  |  |  |  |  |  | NM_001171933.1 variant 6 | NP_001165404.1 isoform 6 | 1 | 310 |
|  |  |  |  |  |  |  |  | NM_001171935.1 variant 8 | NP_001165406.1 isoform 8 | 1 | 481 |
| 11 | *USH1C* | NM_153676.3 variant b3 | NP_710142.1 isoform b3 | 27 | 3246 | 2700 | 899 | NM_005709.3 variant 1 | NP_005700.2 isoform a | 1 | 75 |
| 11 | *MYO7A* | NM_000260.3 variant 1 | NP_000251.3 isoform 1 | 49 | 7465 | 6648 | 2215 | NM_001127179.2 variant 3 | NP_001120651.2 isoform 3 | 1 | 410 |
| 17 | *USH1G* | NM_173477.2 | NP_775748.2 | 3 | 3561 | 1386 | 461 |  |  |  |  |
| 9 | *DFNB31* | NM_015404.3 variant 1 | NP_619636.2 isoform 1 | 12 | 4079 | 2724 | 907 | NM_001083885.2 variant 2 | NP_001077354.2 isoform 2 | 1 | 110 |
| 10 | *PDZD7* | NM_001195263.1 variant 1 | NP_001182192.1 isoform 1 | 17 | 4164 | 3102 | 1033 | NM_024895.4 variant 2 | NP_079171.1 isoform 2 | 1 | 285 |
| 9 | *TMC1* | NM_138691.2 | NP_619636.2 | 24 | 3201 | 2283 | 760 |  |  |  |  |
| 11 | *TECTA* | NM_005422.2 | NP_005413.2 | 23 | 6468 | 6468 | 2155 |  |  |  |  |
| 12 | *VEZT* | NM_017599.3 variant 1 | NP_060069.3 isoform 1 | 12 | 4580 | 2340 | 779 | NR_038242.1 variant 2 | non coding | 1 | 74 |
| 13 | *GJB2* | NM_004004.5 | NP_003995.2 | 2 | 2347 | 681 | 226 |  |  |  |  |
| 13 | *GJB6* | NM_001110219.2 variant 1 | NP_001103689.1 isoform 1 | 5 | 2178 | 786 | 261 | NM_006783.4 variant 3 | NP_006774.2 isoform1 | 1 | 369 |
| 17 | *MYO15A* | NM_016239.3 | NP_057323.3 | 65 | 11876 | 10593 | 3530 |  |  |  |  |
| X | *CHM* | NM_000390.2 variant 1 | NP_000381.1 isoform a | 15 | 5442 | 1962 | 653 | NM_001145414.1 variant 2 | NP_001138886.1 isoform b | 1 | 2493 |
|  |  |  |  |  |  |  |  |  |  |  |  |
| **Table S2:** Table of 19 human genes targeted for the next-generation sequencing. Additional exon(s) refers to the number of exons differing from the main isoform. | | | | | | | | | | | |
